# *HES4* is a potential biomarker for bladder cancer: a Mendelian randomization study

**DOI:** 10.7150/jca.92657

**Published:** 2024-01-21

**Authors:** Zhiyong Tan, Xiaorong Chen, Haihao Li, Yinglong Huang, Shi Fu, Mingxia Ding, Jiansong Wang, Haifeng Wang

**Affiliations:** 1 Department of Urology, The Second Affiliated Hospital of Kunming Medical University, No. 347, Dianmian Street, Wuhua District, Kunming, 650101, Yunnan, People's Republic of China.; 2 Urological disease clinical medical center of Yunnan province, The Second Affiliated Hospital of Kunming Medical University, No. 347, Dianmian Street, Wuhua District, Kunming, 650101, Yunnan, People's Republic of China.; 3 Scientific and Technological Innovation Team of Basic and Clinical Research of Bladder Cancer in Yunnan Universities, The Second Affiliated Hospital of Kunming Medical University, No. 347, Dianmian Street, Wuhua District, Kunming, 650101, Yunnan, People's Republic of China.; 4 Department of Kidney Transplantation, The Third Hospital of Sun Yat-Sen University, Guangzhou, People's Republic of China.

**Keywords:** Bladder cancer, Mendelian randomization, Biomarker, Tumor immune microenvironment

## Abstract

**Background:** Patients with bladder cancer (BLCA) have a poor prognosis and little progress has been made in treatment. Therefore, the purpose of this work was to employ Mendelian randomization (MR) and transcriptome analysis to identify a novel biomarker that could be used to reliably diagnose BLCA.

**Methods:** TCGA-BLCA and GSE121711 datasets were obtained from public databases. Genome-wide association study (GWAS) data of BLCA outcome (373,295 samples containing 9,904,926 single nucleotide polymorphisms) were obtained through the IEU OpenGWAS database. Differentially expressed genes were applied as exposure factors, and MR analysis was performed to identify genes that had a causal relationship with BLCA. Then, the patients were divided into high and low expression groups according to the expression levels of candidate genes, and genes with survival differences were identified. Univariate and multivariate Cox regression were used to investigate the prognostic value of the expression of these genes. A nomogram was constructed based on independent prognostic factors, and we analyzed the functions and pathways associated with the identified genes as well as their relationship with the immune microenvironment.

**Results:** *HES4* was identified as a biomarker. *HES4* status, age, and stage were identified as independent prognostic factors, and an excellent nomogram was established. Bioinformatic analysis suggested that *HES4* might be associated with the activation of the immune response, bone development, and cancer pathways. The BLCA samples were divided into high and low *HES4* groups. The stromal score and 33 immune cells were remarkably different between the two groups, with *HES4* expression being negatively correlated with macrophages and mast cells, and positively correlated with eosinophils and central memory CD4+ T cells. Finally, *HES4* was up-regulated in cancer samples in both TCGA-BLCA and GSE121711 datasets.

**Conclusion:** This study identified *HES4* as an independent prognostic factor for BLCA outcome based on MR and transcriptome analysis, which provides useful information for future research on and treatment of BLCA.

## 1. Introduction

Bladder cancer (BLCA) is a common malignant tumor of the urological system. It occurs more frequently in men for whom it is the sixth most prevalent cancer 1. Currently identified risk factors for BLCA are smoking and occupational exposures. Recent studies have revealed that specific dietary factors, microbiota imbalances, gene-external risk factor interactions, exposure to diesel exhaust emissions, and pelvic radiotherapy may also be contributing factors 2. Depending on the depth of tumor infiltration, it can be classified as non-muscle invasive bladder cancer (NMIBC) and muscle-invasive bladder cancer (MIBC). Approximately 75% 75% of patients have NMIBC at the time of the initial visit 3, and the 5-year overall survival (OS) rate is nearly 90% 4. Approximately 10-30% of NMIBC patients progress to MIBC with their 5-year OS decreasing to 60-70% 4,5, and 10% of these patients develop metastasis beyond the bladder, decreasing the 5-year OS to 5-30%. Apart from traditional therapies involving surgery, chemotherapy, and radiotherapy, immunotherapy has started to be used for the treatment of patients with advanced BLCA. However, only a limited number of patients benefit from immunotherapy 6, with one of the crucial reasons for this being the lack of biomarkers available to guide diagnosis and treatment. Although Chen et al. identified urinary tumor DNA methylation assessment as a novel method for early diagnosis of BLCA 7, it is still urgent to study the underlying molecular mechanisms of BLCA occurrence and progression in order to identify more sensitive and precise biomarkers. This will facilitate the early diagnosis and treatment of BLCA, which will help to increase the survival rate and improve the prognosis of patients.

Mendelian randomization (MR) uses genetic variants as instrumental variables (IVs), and the random assignment of effect alleles is similar to the randomization process in randomized controlled trials, which strengthens the inference of causality between exposure and outcome 8-10. The strength of MR is that the method minimizes the interference of confounding factors such as environment and adaptation, and also reduces reverse causality since the genetic variation used to substitute for the effect of exposure cannot be changed in turn by the occurrence and progression of the outcome 11,12. The association of coffee intake, alcohol consumption, smoking, human papillomavirus (HPV) infection, and benign prostatic hyperplasia (BPH) with the development of BLCA has been investigated in previous MR studies, with results suggesting that smoking, HPV infection, and BPH may be causally associated with BLCA, whereas coffee intake and alcohol consumption are not 13-16. The European Association of Urology stated that there is currently insufficient evidence to support the use of tumor mutation burden (TMB), gene expression signature or molecular typing in the management of patients with BLCA 17. Transcriptomic MR studies may help to change this by identifying genes causally associated with BLCA.

In this study, MR analysis of the BLCA transcriptome was performed to identify novel causal BLCA genes. The molecular mechanisms by which these genes might impact BLCA development and progression were investigated by enrichment analysis, ceRNA analysis, immune infiltration analysis, and targeted drug prediction.

## 2. Materials and methods

### 2.1 Data sources

The Cancer Genome Atlas (TCGA) bladder cancer dataset (TCGA-BLCA) was obtained from the TCGA database (https://www.cancer.gov/tcga), which contained 397 BLCA samples with complete survival information. GSE121711 (GPL17586) was obtained from the Gene Expression Omnibus (GEO) database (https://www.ncbi.nlm.nih.gov/geo), which contained 8 BLCA samples and 10 normal samples. Genome-wide association study (GWAS) data of BLCA outcomes (ieu-b-4874; 373,295 samples containing 9,904,926 single nucleotide polymorphisms (SNPs)) and independent SNPs were obtained through the IEU OpenGWAS database (https://gwas.mrcieu.ac.uk/).

### 2.2 Differentially expressed analysis

Differentially-expressed genes (DEGs) were obtained between tumor and normal samples using DESeq2 (v3.44.3) in the R package 18. An adjusted *P* < 0.05 and |log_2_FC| > 1 were used as cutoffs to define differential expression. Univariate Cox regression was used to identify DEGs associated with outcome, with *P* < 0.05 considered statistically significant. Gene Ontology (GO) and Kyoto Encyclopedia of Genes and Genomes (KEGG) was used to explore the functions and associated pathways of the identified genes using clusterProfiler (v3.16.0) in R 19.

### 2.3 Selecting IVs for MR analysis

To ensure valid MR analysis, three basic assumptions should be satisfied: (1) IVs are closely related to exposure factors, (2) IVs cannot be associated with any confounding factors, and (3) exposure is the only way that genetic variation can affect the outcome. For univariate MR analysis, SNPs were used as IVs. The R package TwoSampleMR (v0.5.6) 20 was used to read the exposure factors and screen the IVs. SNPs were selected using the genome-wide significance threshold *P* < 5×10^-8^. Appropriate SNPs were then retained using a linkage imbalance threshold of r^2^ = 0.001 in the Euro 1000 genome reference panel. Finally, SNPs that were associated with the results but with *P* < 5×10^-8^ were excluded 21. Palindromic SNPs with intermediate allele frequencies were eliminated when combining exposure and outcome datasets.

### 2.4 MR analysis

MR analysis was used to explore the causal effect between exposure factors (DEGs identified by the Cox analysis) and outcome (BLCA). In this study, the MR-Egger, weighted median, inverse variance weighted (IVW), simple mode, and weighted mode 22 methods were used for univariate MR analysis. The IVW method was used as the main MR analysis to identify genes that had a causal relationship with BLCA. A P value cutoff of 0.05, was used to identify causal relationships. A scatter plot was used to determine the correlation between exposure factors and outcome, a forest plot was used to determine the predictive exposure factors of each SNP site for the diagnosis of the outcome, and a funnel plot was used to evaluate whether MR was in line with Mendel's second law of randomization.

### 2.5 Sensitivity analysis

Sensitivity analysis was used to evaluate the reliability of MR analysis. Firstly, the Q value of the heterogeneity test was more than 0.05, indicating that there was no heterogeneity. Secondly, the P-value of the horizontal pleiotropy test was greater than 0.05, indicating that there were no confounding factors. Finally, the leave-one-out (LOO) test was used to determine whether the remaining SNPs had an impact on the overall analysis, even if one SNP was removed.

### 2.6 Identification of key genes

Univariate Cox regression and multivariate Cox regression were used to analyze the genes that had a causal relationship with BLCA, and genes whose Mendel risk values and univariate Cox risk values were in the same direction were selected as candidate genes for subsequent analysis. The expression levels of these genes and survival information of cancer patients were extracted from the TCGA dataset for analysis of overall survival (OS), and Kaplan-Meier (K-M) survival curves were constructed. Patients were divided into high and low expression groups based on the expression level of the candidate gene. Candidate genes with significant differences in survival were selected as biomarkers for subsequent analysis.

### 2.7 Independent prognostic analysis

Clinicopathological factors and biomarker expression levels in TCGA-BLCA samples were used to explore the independent prognostic value of the biomarkers by univariate Cox analysis. A nomogram was constructed based on significant factors, and patient survival at 1, 3, and 5 years was predicted based on the total score, with the higher the score, the lower the survival. Calibration curve and decision curve analysis (DCA) were used to evaluate the predictive ability of the nomogram.

### 2.8 Single gene GSEA and co-expression gene enrichment analysis

To explore the regulatory pathways and biological functions related to biomarkers expression, a single gene GSEA analysis was performed. Firstly, the correlation coefficients between the expression levels of all genes and biomarkers were calculated as the sequencing criteria, and GSEA enrichment analysis was performed using clusterProfiler (v3.16.0) 23 in the R package. The screening criteria were |NES| > 1, adj. *P* < 0.05. To further understand the functions and pathways associated with genes that were co-expressed with the biomarkers, LinkedOmics was used to identify co-expressed genes, their correlation was assessed using Spearman analysis, and GO and KEGG analyses were conducted. The screening criterion was adj. *P* < 0.05.

### 2.9 Estimation of tumor immune microenvironment (TIME)

A series of correlation analyses were used to assess the differences in the TIME between high and low expression groups. The R package estimate (v1.0.13) 24 was used to calculate the immune score, matrix score, ESTIMATE score, and tumor purity of each sample, and the association between them was examined by calculating the Spearman correlation coefficient. Seven different approaches were used to assess immune cell differences between high and low expression groups (TIMER, CIBERSORT, CIBERSORT-ABS, quantTIseq, MCP-counter, xCELL, and EPIC). TIDE is a computational method for predicting immune checkpoint blocking (ICB) responses 25. From the RNA expression profile, TIDE prediction scores were calculated to predict the likelihood that BLCA patients would respond to immunotherapy. The lower the TIDE score, the lower the likelihood of immune escape. In addition, subMap analysis was performed to predict differences in response to anti-PD-1 or anti-CTLA-4 immunotherapy between high and low expression groups. Finally, as immune checkpoint inhibitors (ICI) are used clinically for cancer immunotherapy, Spearman correlation analysis was used to investigate the relationship between the identified biomarkers and expression of genes associated with immune checkpoints.

### 2.10 Prediction of chemotherapy drug sensitivity and molecular docking

To attempt to identify high-potency anti-BLCA drugs, the R pRRopheticPredict package (v5.0) was used to calculate the half-inhibitory concentrations (IC_50_) of 138 drugs in the CGP database. The difference in the IC_50_ values between the high and low expression groups was then calculated. Spearman correlation analysis was used to assess the correlation between the IC_50_ values and the expression of the identified biomarkers. To identify a potential novel therapeutic target for an identified drug, it was docked to the structure of HES4. The protein structure was obtained from the PDB 26, and all small molecules (including water) were removed. Protein hydrogenation and charge calculations were performed using AutoDock. Drug structures were retrieved from the PubChem database (https://pubchem.ncbi.nlm.nih.gov) 27 and charge-balanced, rotatable bond checks were performed using AutoDock. A range of docking boxes were selected based on the receptor active site. AutoDock vina was used to perform protein-ligand docking, and the structure with the lowest binding free energy was selected. PyMol software was used for visualization.

### 2.11 Evaluation of genetic mutations, tumor mutation burden (TMB), and copy number variation (CNV)

To identify the gene mutations in the high and low expression group, somatic mutation data were downloaded from the Genomic Data Commons (GDC) database 28, and mutations in the high and low expression groups were identified using maftools (v2.4.10) 29 in R. TMB is a quantitative biomarker that reflects the total number of mutations carried by tumor cells. To investigate differences in TMB between the high and low expression groups, we calculated TMB values and made boxplots. CNV in the TCGA-BLCA data were displayed on chromosome plots, and Genomic Identification of Significant Targets in Cancer (GISTIC) was used to analyze copy number loss or gain in all samples followed by a Wilcoxon test between the high and low expression groups to identify statistically significant differences.

### 2.12 Construction of a competing endogenous RNA (ceRNA) network

The miRWalk tool (http://mirwalk.umm.uni-heidelberg.de/) was used to predict biomarker-related micro RNAs (miRNAs). miRNA-related long non-coding RNAs (lncRNAs) were identified in Starbase (http://starbase.sysu.edu.cn/) using a threshold of clipExpNum > 1, and the ceRNA network was constructed.

### 2.13 Expression verification of biomarkers

The expression levels of key genes were verified in the TCGA-BLCA and GSE121711 datasets, and results were displayed using boxplots.

### 2.14 Statistical analysis

Statistical analysis was performed using R software (v4.1.0). Differences between groups were analyzed using Wilcox tests and t-tests. *P‐value < 0.05; **P‐value < 0.005; ***P‐value < 0.0005; ****P‐value < 0.00005, represented a significant difference. In the GSEA, the thresholds used were |NES| > 1 and adj. *P* < 0.05.

## 3. Results

### 3.1 Identification of DEGs with differences in risk ratio

**Figure 1:**
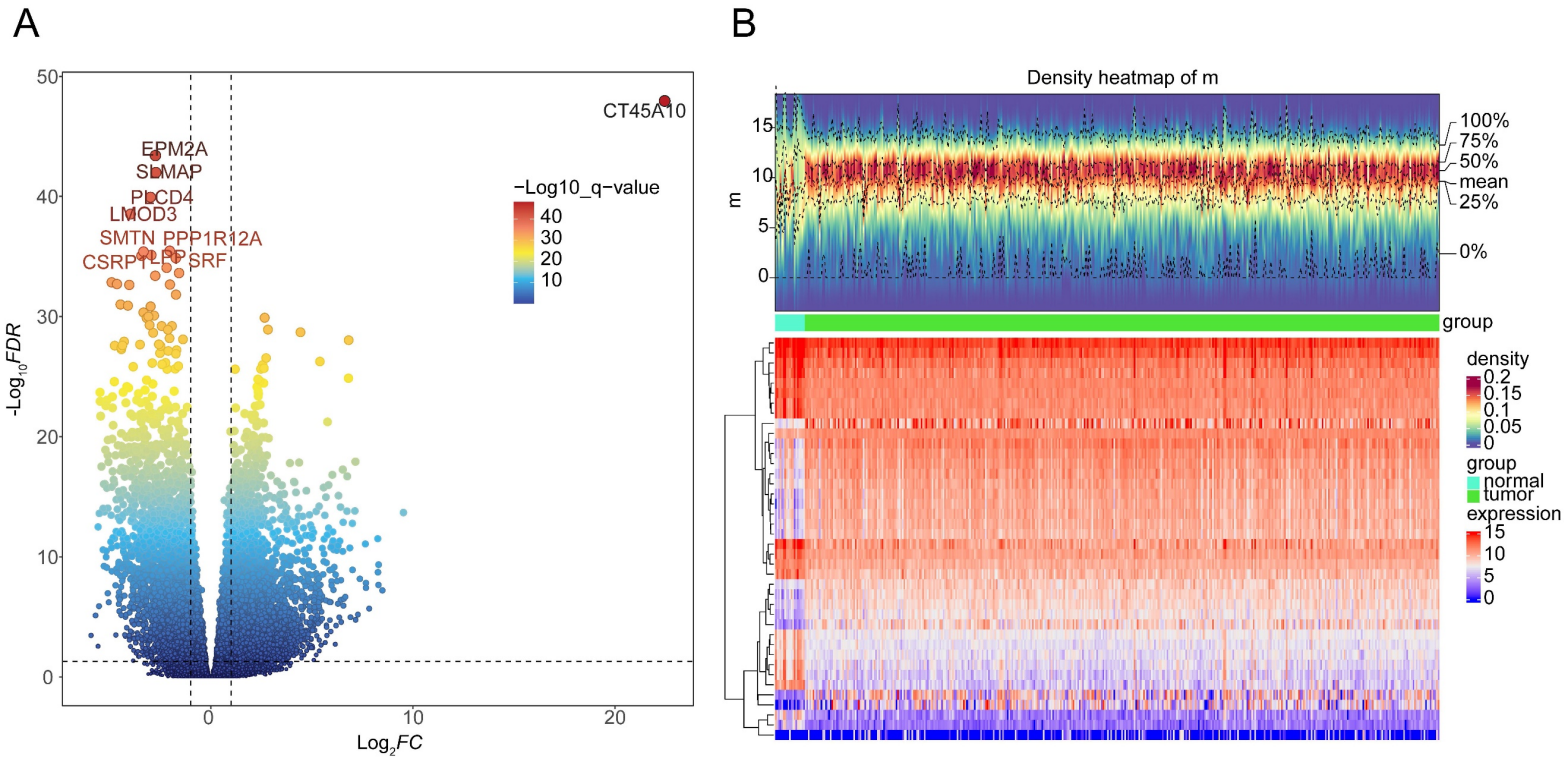
Identification of differentially expressed genes (DEGs) between BLCA and normal groups in the TCGA-BLCA dataset. **A** Volcano plot and **B** heatmap (density) plot illustrating the expression of 8467 DEGs.

A total of 8467 DEGs were identified between BLCA and normal samples, with 3374 down-regulated and 5093 up-regulated **(Figure 1A-B)**. Univariate Cox proportional hazard regression analysis was performed on all 8467 DEGs, with a total of 3678 giving significant differences in survival (*P* < 0.05) **(Supplementary Table 1)**.

### 3.2 Functional enrichment and pathway analysis

**Figure 2:**
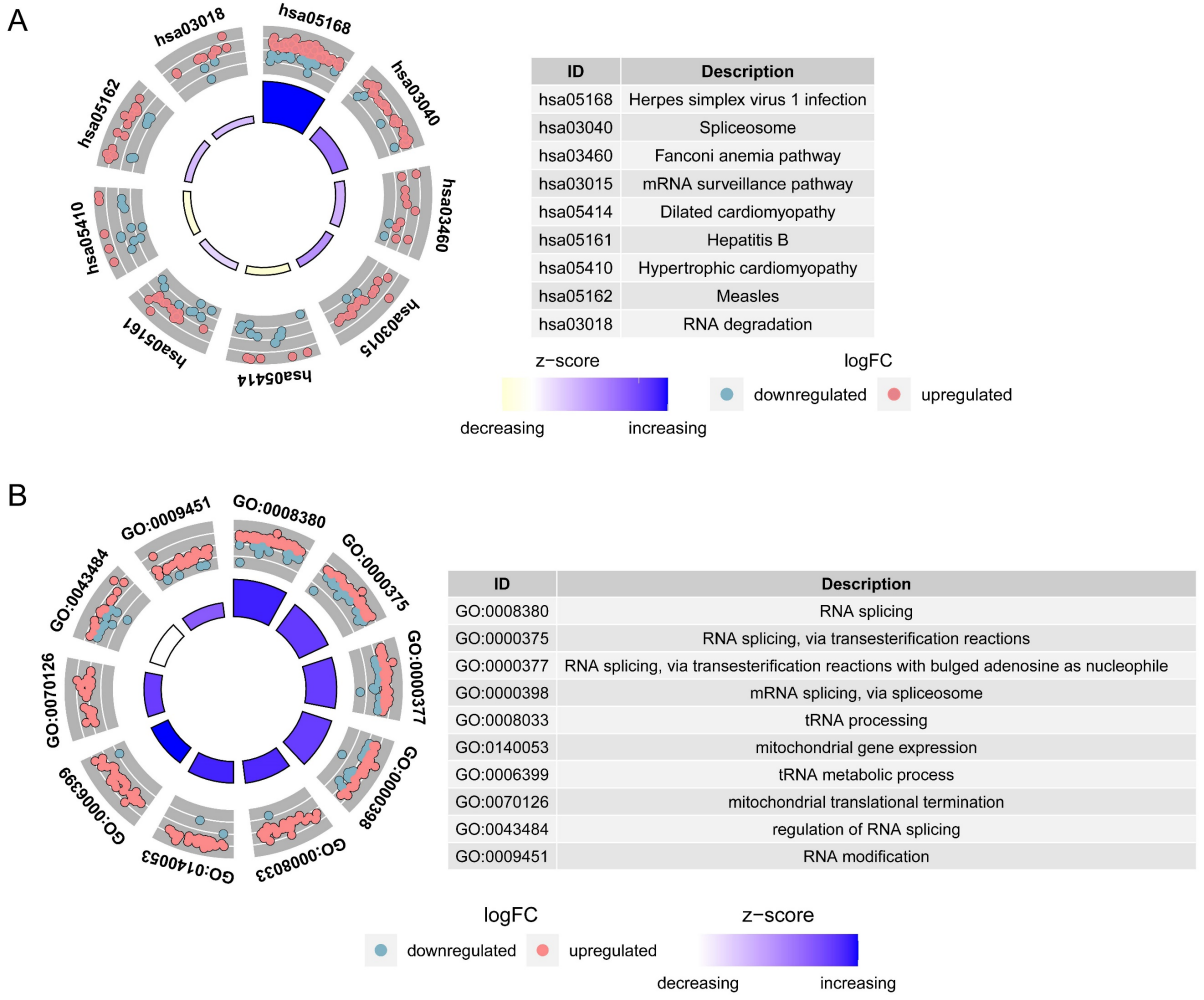
Functional enrichment circle plots of 3678 DEGs with significant differences in survival in univariate Cox analysis. **A** Kyoto Encyclopedia of Genes and Genomes (KEGG) enrichment analysis. **B** Gene Ontology (GO) enrichment analysis.

A total of 9 KEGG pathways and 58 GO function entries were enriched in the set of 3678 genes identified above. The most significant KEGG pathways were herpes simplex virus infection, spliceosome, Fanconi anemia pathway, mRNA surveillance pathway, hypertrophic cardiomyopathy and RNA degradation **(Figure 2A)**. The most significant GO terms were RNA splicing via spliceosome and tRNA processing **(Figure 2B)**.

### 3.3 Identification of genes with a causal relationship with BLCA

**Figure 3:**
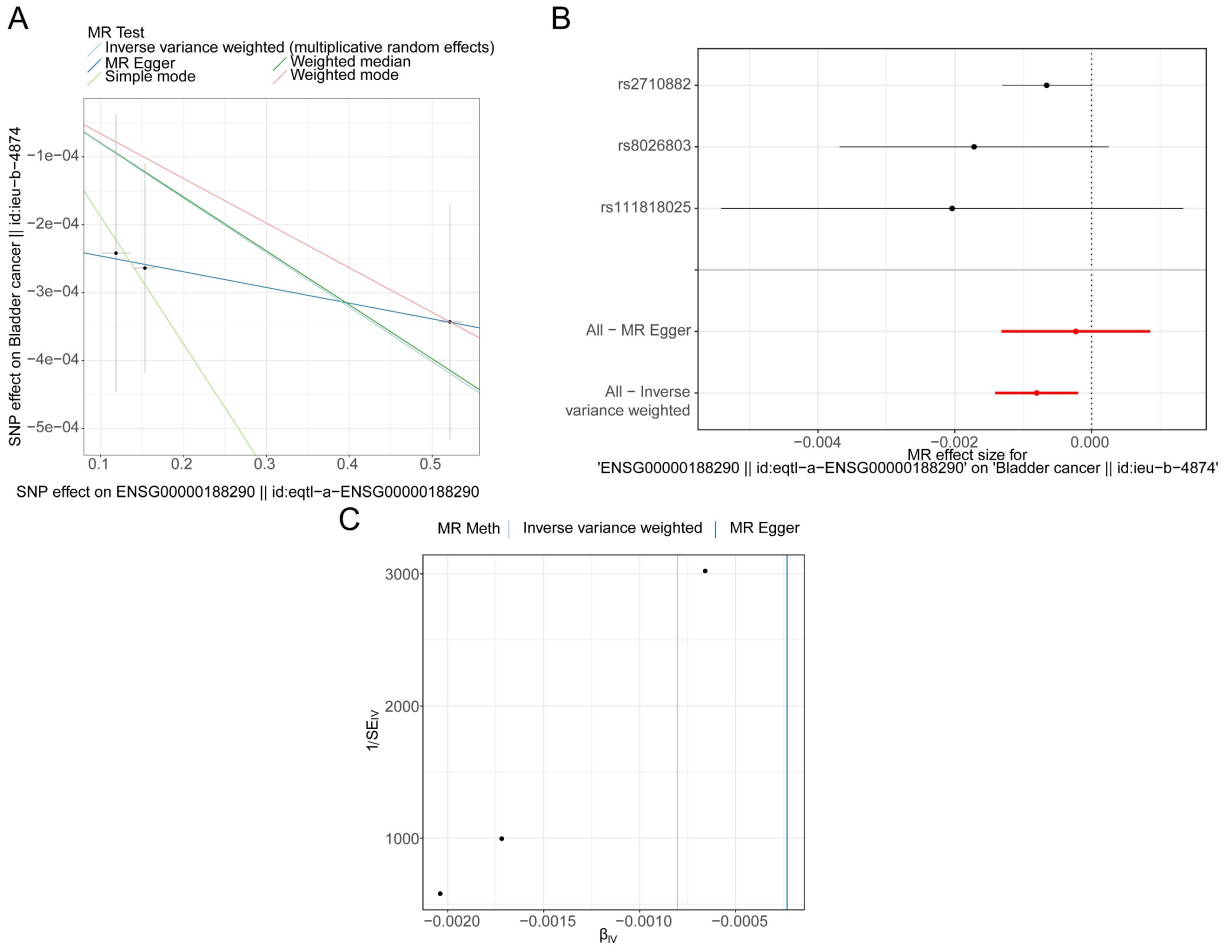
*HES4* has a causal relationship with BLCA. **A** Scatter plots for the relationship of *HES4* expression and BLCA. **B** Forest plot for the efficacy of *HES4* in predicting BLCA risk. **C** Funnel plot demonstrating a symmetrical distribution of single nucleotide polymorphisms of *HES4* in both inverse variance weighted and MR-Egger algorithms.

The IVW results showed that 8 genes were causally associated with BLCA. These were *NSUN5P2* (*P* = 0.025), *GHRLOS* (*P* = 0.000), *TPM3P9* (*P* = 0.021), *HES4* (*P* = 0.021), *BATF2* (*P* = 0.002), *PLXNA4* (*P* = 0.019), *TGFBI* (*P* = 0.014) and *EPHB1* (*P* = 0.040). *HES4* (Odds ratio [OR] = 0.888), *PLXNA4* (OR = 0.919), and *EPHB1* (OR = 0.811) were safety factors for BLCA, and *NSUN5P2* (OR = 1.030), *GHRLOS* (OR = 1.011), *TPM3P9* (OR = 1.018), *BATF2* (OR = 1.046), and *TGFBI* (OR = 1.074) were risk factors **(Supplementary Table 2)**. For *HES4*, the scatter plot shows that the IVW slope is negative with no intercept **(Figure 3A)**. The forest map also demonstrates that *HES4* is a safety factor **(Figure 3B)**. SNPs were randomly distributed on both sides of the IVW line, indicating that MR conformed to Mendel's second law **(Figure 3C)**. The results for all other genes are shown in **Supplementary Figure 1-3**.

### 3.4 Identification of *HES4* as biomarker

**Figure 4:**
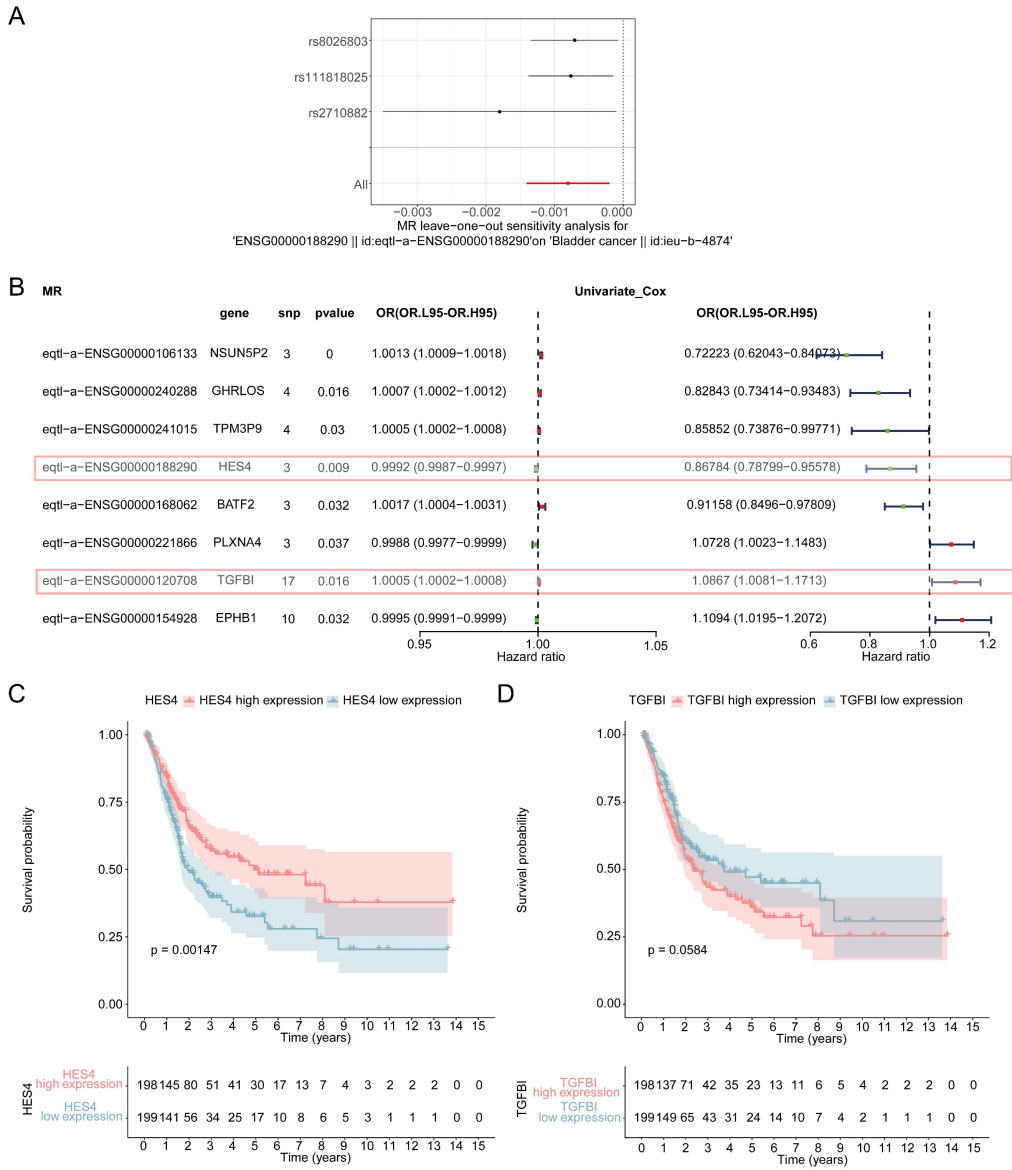
Sensitivity analysis of MR. **A** Forest plot of leave-one-out analysis of the relationship of *HES4* and BLCA. **B** Forest plot for the univariate Cox analysis of eight genes with a causal relationship with BLCA. **C** Kaplan-Meier (K-M) curves of high and low *HES4* expression groups. **D** K-M curve of high and low *TGFBI* expression groups.

Sensitivity analysis was used to assess the reliability of the MR results. The heterogeneity test demonstrated that there was no heterogeneity in the analysis (*P* < 0.05) **(Table 1)**, the horizontal pleiotropy test showed that there were no confounding factors in the study (*P* > 0.05) **(Table 2)**, and LOO revealed that there were no outlier SNPs, all of which suggest that the MR results were reliable **(Figure 4A, Supplementary Figure 4)**. The forest maps of these 8 genes showed that the Mendelian risk values of *HES4* (MR: *P* = 0.009, OR = 0.9992; univariate Cox analysis: OR = 0.868) and *TGFBI* (MR: *P* = 0.016, OR = 1.0005; univariate Cox analysis: OR = 1.0867) were in the same direction as the univariate Cox risk values **(Figure 4B)**. Finally, OS analysis was performed using the expression levels of *HES4* and *TGFBI* and the patient survival information **(Figure 4C-D)**. *HES4,* with the more significant impact on survival prognosis, was selected to proceed with further analyses.

### 3.5 *HES4*, age, and stage are independent prognostic factors

**Figure 5:**
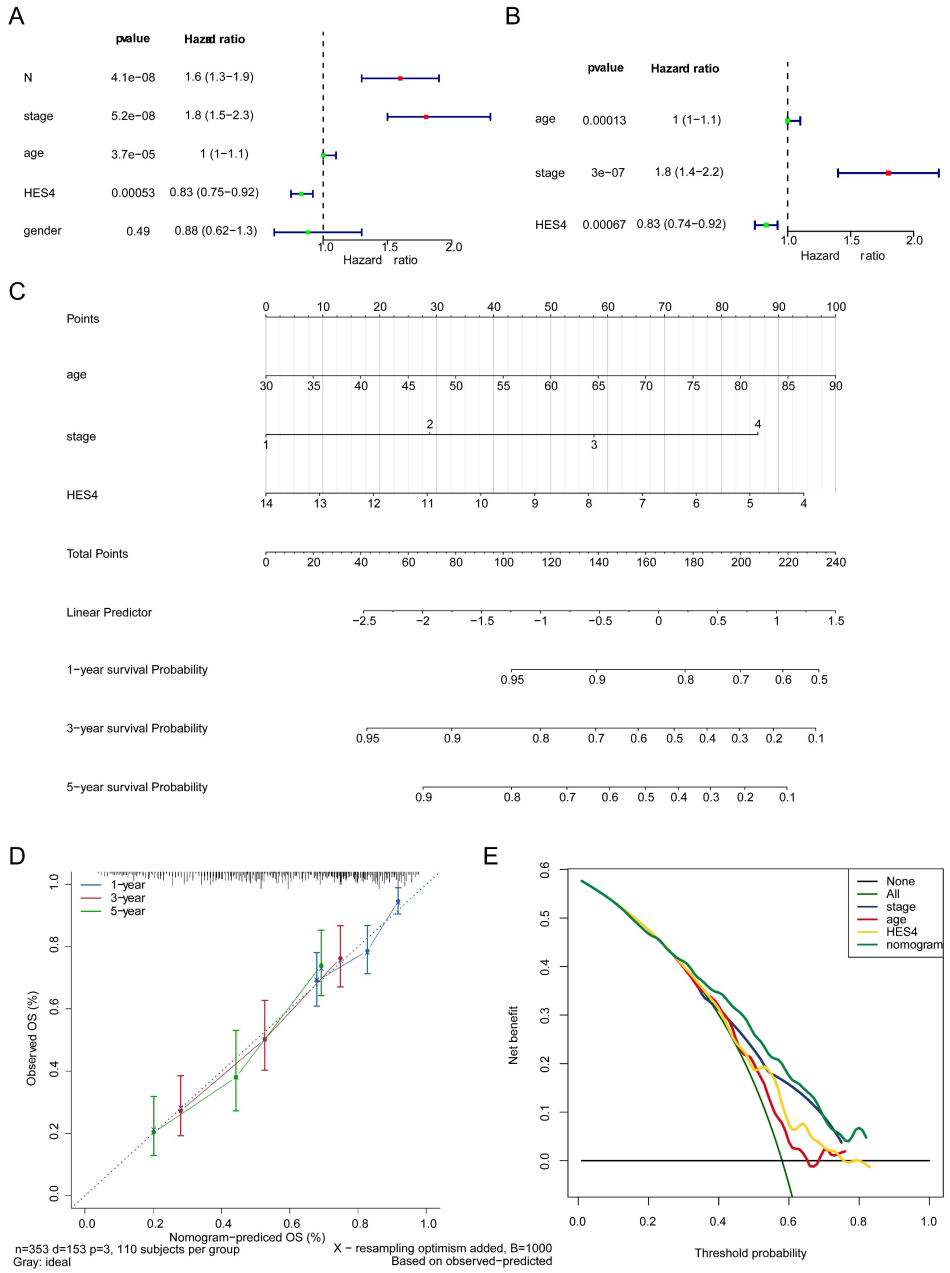
Independent prognostic value of *HES4*. Forest plot of **A** univariate and **B** multivariate Cox analyses to identify independent prognostic factors in TCGA-BLCA samples. **C** Nomogram based on *HES4* expression, stage, and age. **D** Nomogram calibration curve. **E** Decision curve analysis of the nomogram and independent prognostic factors.

The clinicopathological factors and *HES4* gene expression of TCGA-BLCA samples were used for Cox analysis. The results indicated that the N (*P* = 4.13×10^-8^), stage (*P* = 5.21×10^-8^), age (*P* = 3.68×10^-5^) and *HES4* expression (*P* = 0.0005) were significant prognostic factors **(Table 3, Figure 5A)**. *HES4* expression, age, and stage had significant *P*-values in multivariate Cox analysis, indicating that they were independent prognostic factors **(Table 4, Figure 5B)**. A nomogram was constructed based on *HES4* expression, clinical factors, and the 1 -, 3 -, and 5-year survival rates **(Figure 5C)**. The calibration curve slope (c-index = 0.677) was close to 1, showing that the nomogram prediction was more accurate. The slopes for 1, 3, and 5 years were 0.6935, 0.2826, and 0.2122, respectively, indicating that the prediction was best at 1 year **(Figure 5D)**. DCA showed a nomogram with the sole advantage of *HES4*, indicating that the nomogram has good forecasting ability** (Figure 5E)**.

### 3.6 Signaling mechanisms related to *HES4*

**Figure 6:**
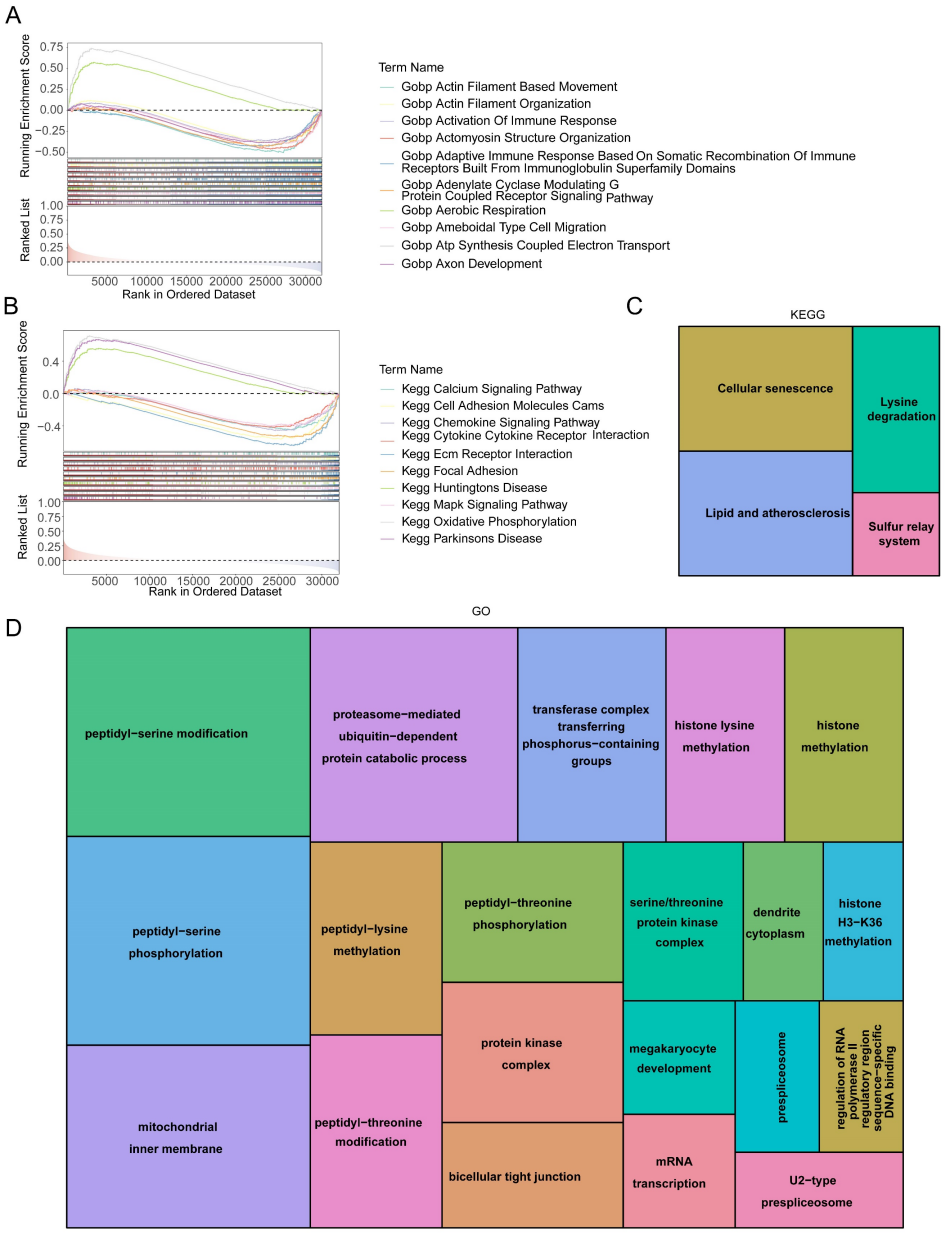
*HES4* function. Gene Set Enrichment Analysis (GSEA) diagram of *HES4* based on **A** GO and **B** KEGG databases. Tree maps for the enriched **C** KEGG and **D** GO terms of 100 genes which were significantly co-expressed with *HES4*.

GSEA enrichment analysis was used to understand the functions and pathways associated with *HES4*. A total of 3822 GO entries contained *HES4*, including actomyosin structural organization, aerobic respiration, ATP synthesis coupled electron transport, and bone development **(Figure 6A)**. 81 KEGG pathways contained *HES4*, including oxidative phosphorylation, Parkinson's disease, and cancer pathway **(Figure 6B)**. Using LinkedOmics and Spearman correlation analysis, the 50 genes whose expression were most strongly positively and negatively correlated with *HES4* were found. Enrichment analysis of these 100 genes identified 4 KEGG pathways and 78 GO entries. The KEGG pathways were cell senescence, lysine degradation, sulfur relay system, and lipid and atherosclerosis. Cellular senescence and lipid and atherosclerosis pathways contained the most genes **(Figure 6C)**. Enriched GO terms included peptidyl-serine modification, proteasome-mediated ubiquitin-dependent protein catabolism, peptidyl-threonine modification, megakaryocyte development, histone methylation, and glycoprotein methylation. Peptidyl-serine modification contained the most genes **(Figure 6D)**.

### 3.7 Differences in the immune microenvironment between high and low expression groups

**Figure 7:**
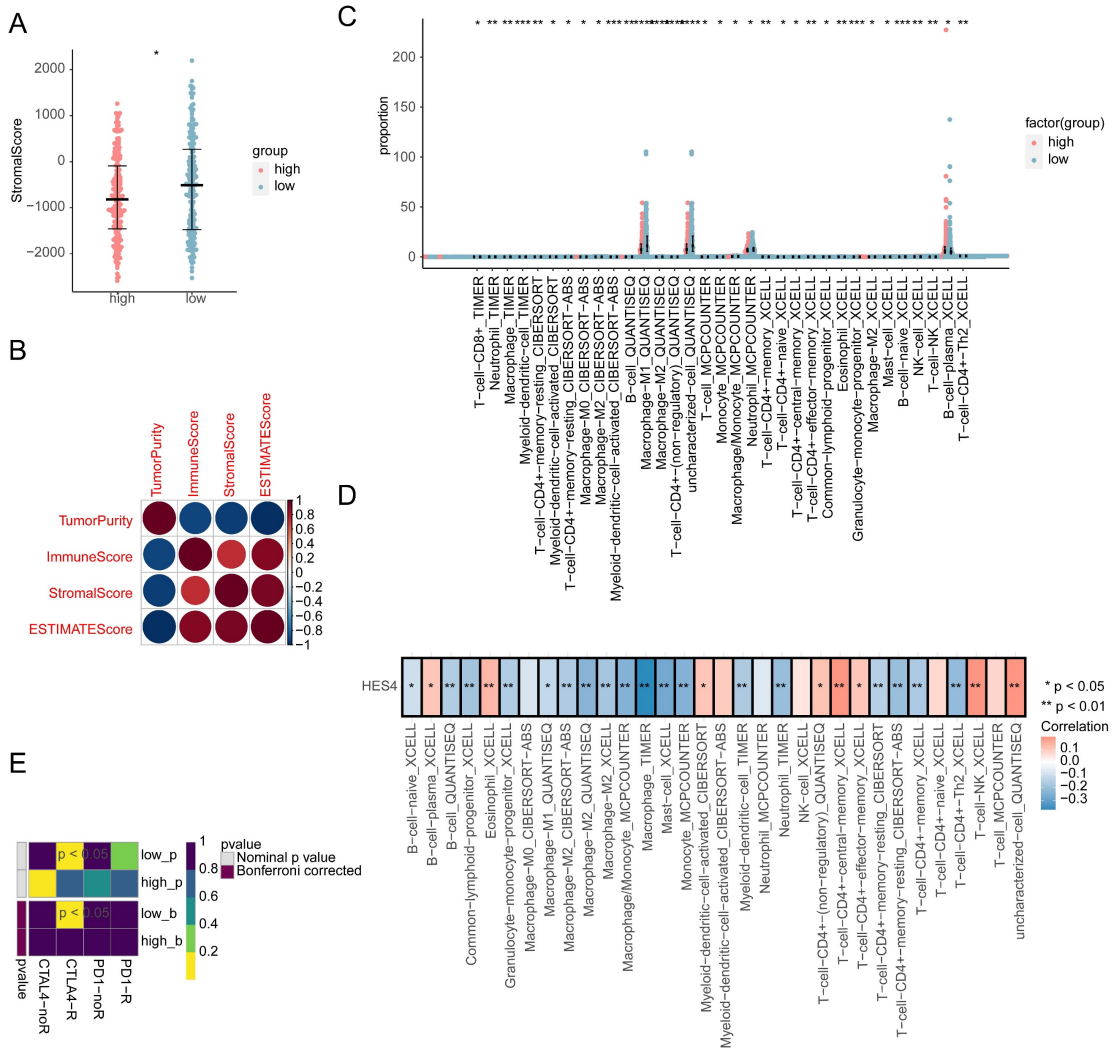
Immune infiltration analysis comparing the high and low *HES4* expression groups. **A** Stromal scores of the different expression groups. **B** Correlation heatmap of tumor purity, immune scores, stromal scores, and ESTIMATE scores. **C** Boxplots for the infiltration levels of 33 immune cells were calculated using different algorithms between high and low expression groups. **D** Correlation heatmap of differentially abundant immune cells and *HES4* expression. **E** SubMap analysis predicted that the high *HES4* expression group was more sensitive to PD-1 therapy.

The immune score, stromal score, ESTIMATE score, and tumor score were calculated for the high and low expression groups. Only the stromal score was significantly different between the two groups, with the low expression group having a higher stromal score **(Figure 7A)**. The correlation between the four scores was explored, and it was found that the tumor purity was significantly negatively correlated with other indicators (stromal score: cor = -0.94, *P* = 1.17×10^-170^; immune score: cor = -0.92, *P* = 2.08×10^-165^; ESTIMATE score: cor = -1.0, *P* = 0), while the correlation between the remaining indicators was significantly positive (correlation of stromal score and immune score was 0.74, *P* = 2.21*10^-71^; correlation of stromal score and ESTIMATE score was 0.94, *P* = 1.17*10^-190^; correlation of immune score and ESTIMATE score was 0.92, *P* = 2.08*10^-165^;** Figure 7B)**. In total, 7 different algorithms were used to evaluate 119 immune cell differences between the high and low expression groups. A total of 33 immune cell types had significantly different abundances between the two groups, with T-cells, monocytes, macrophage/monocyte and neutrophils cells having a higher abundance **(Figure 7C)**. The abundance of 8 cell types was significantly positively correlated with *HES4* expression, including central memory CD4+ T cells and NK T-cells, and the abundance of 18 cell types was significantly negatively correlated with *HES4* expression, including macrophages and mast cell **(Figure 7D)**. TIDE analysis showed no significant differences between the high and low expression groups **(Supplementary Figure 5A)**, and SubMap analysis indicated that the high *HES4* expression group was likely to be more sensitive to PD-1 therapy **(Figure 7E)**. Nine further immune checkpoint molecules were not significantly different between the *HES4* expression groups **(Supplementary Figure 5B)**.

### 3.8 Predicted AZD6482 sensitivity was correlated with *HES4* expression

**Figure 8:**
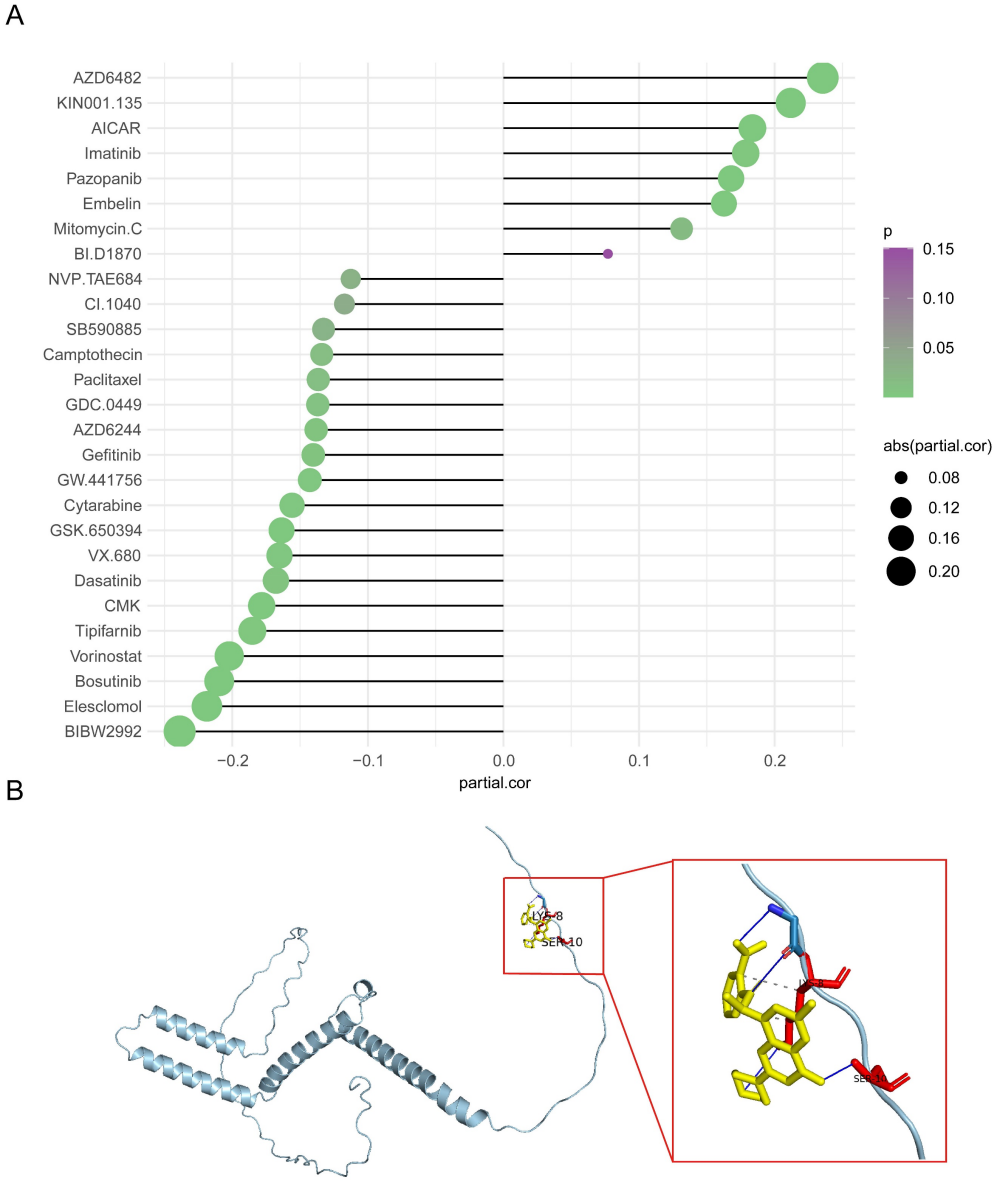
Drug sensitivity analysis in high and low *HES4* expression groups. **A** Correlation lollipop chart of the IC_50_ values of 27 drugs and *HES4* expression. **B** Molecular docking of AZD6482 to HES4.

The IC_50_ values of 138 drugs were predicted in the two *HES4* expression groups. 27 drugs had significantly different IC_50_ values between the two groups, with most drugs having a higher IC_50_ in the low expression group **(Supplementary Figure 6)**. The correlation between *HES4* expression and the IC_50_ values of these 27 drugs was investigated, with 8 drugs having an IC_50_ value positively correlated with *HES4* expression, including AZD6482 and KIN001.135, and 19 drugs having an IC_50_ value negatively correlated with *HES4* expression, including NVP.TAE684 and CI.1040 **(Figure 8A)**. To further understand the potential therapeutic targets of the drug, AZD6482, which greatest correlation and significant difference between the high and low expression groups, was selected and docked to the HES4 structure. As shown in **Figure 8B**, in the lowest energy docked structure, LYS-8 and SER-10 residues had hydrogen bonding interactions with AZD6482. The docking energy between AZD6482 and HES4 was -4.0 kcal/mol.

### 3.9 Assessment of mutations in the high and low *HES4* expression groups

**Figure 9:**
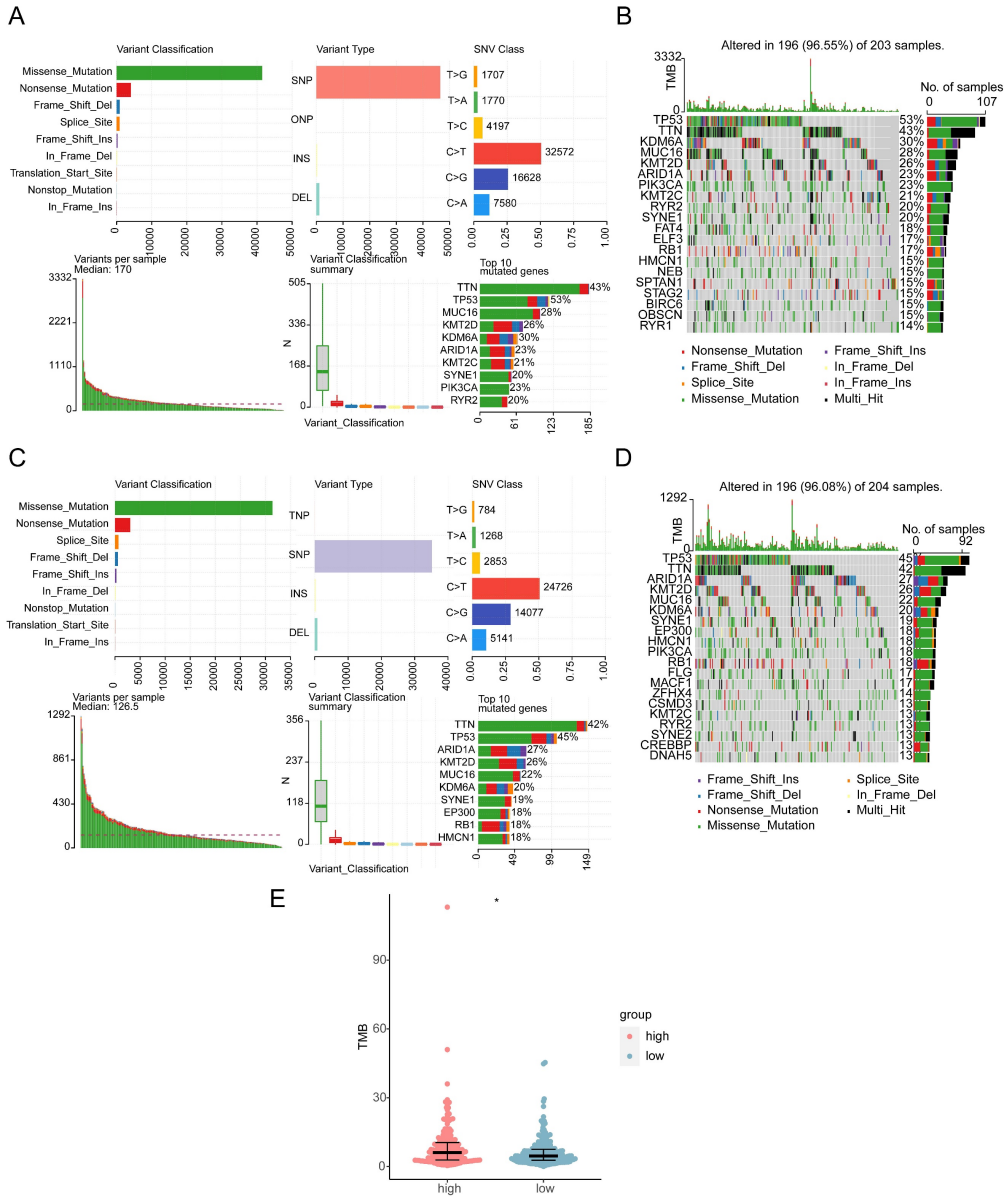
Mutational landscape and tumor mutation burden (TMB) in high and low *HES4* expression groups. **A** Overview of mutations in the high-risk subset of the TCGA-BLCA cohort. **B** Waterfall plot of eight specific mutation profiles of 203 patients with BLCA in the high-risk groups. The bar chart on the right shows the proportion of different mutation types of each gene, and the percentage indicates the mutation frequency of the gene. **C** Overview of mutations in the low-risk subset of the TCGA-BLCA cohort. **D** Waterfall plot of seven specific mutation profiles of 204 patients with BLCA in the low-risk groups. **E** Box plot of TMB in the different risk groups (Wilcoxon test, *p< 0.05).

Missense, frame shift deletion, splice site, frame shift insertion, nonsense, in-frame deletion, and multi-hit mutations were detected in the BLCA genomic data. The genes with the most mutations in both groups were *TP53* and *TTN*. Other genes were mutated at lower frequency, including *RYR2* and *RB1* (**Figure 9A-D**). The high expression group had higher TMB, indicating that the tumor cells in this group had higher neoantigen levels **(Figure 9E)**. Finally, CNV was investigated. CNV location was displayed on chromosome maps** (Supplementary Figure 7A)**, and both copy number amplification and deletion were strongly correlated with BLCA **(Supplementary Figure 7B-C)**. CNV was significantly higher in the low expression group **(Supplementary Figure 7D)**.

### 3.10 Regulatory and ceRNA network analysis of *HES4*

**Figure 10:**
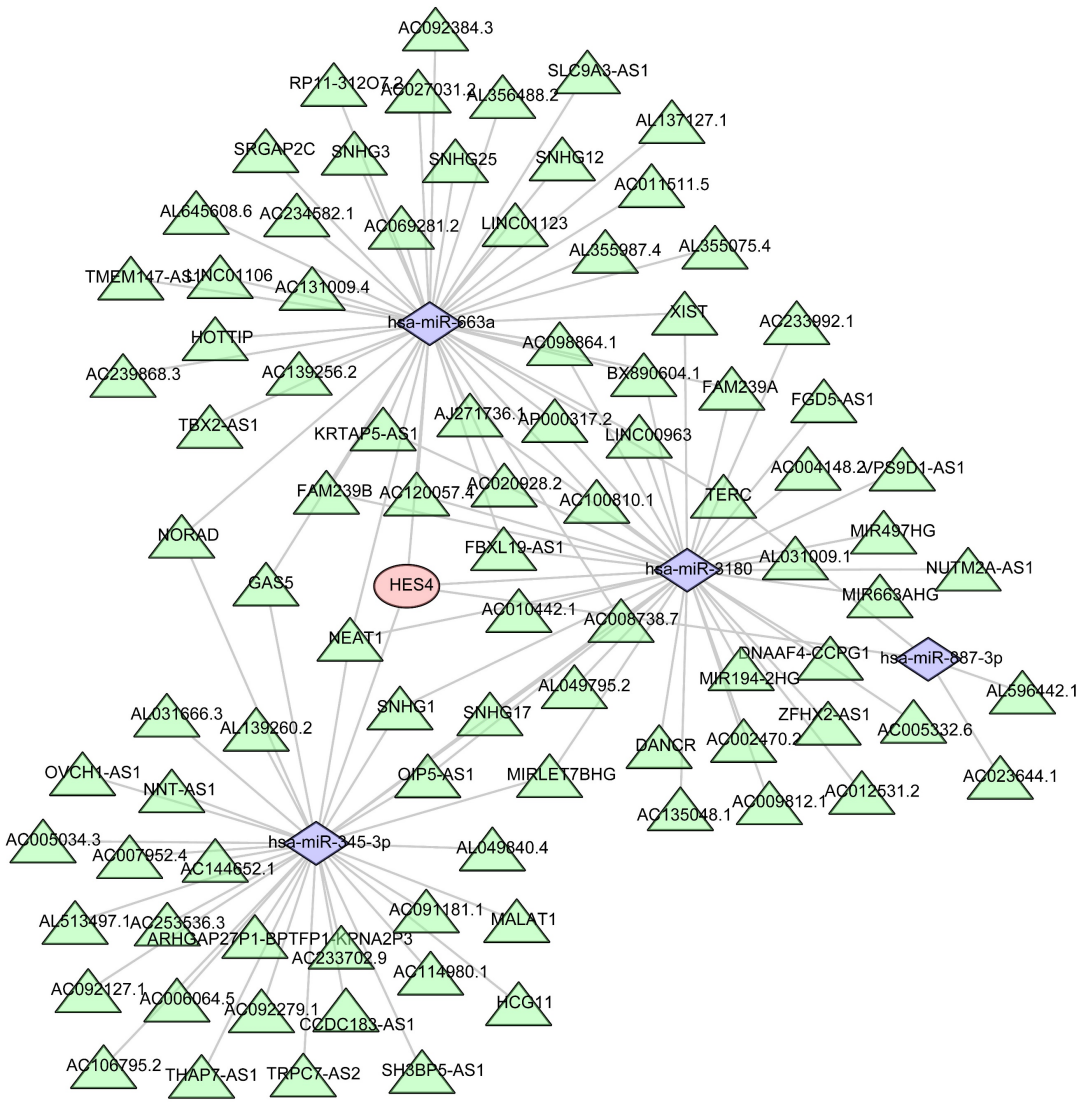
Prediction of the mRNA-miRNA-lncRNA regulatory network of *HES4*. Red represents *HES4*, purple represents miRNA, and green represents lncRNA.

Four miRNAs were predicted to be associated with *HES4* using miRWalk, and 91 lncRNAs associated with 4 miRNAs were predicted using Starbase. Finally, *HES4*, the 4 miRNAs, and the 91 lncRNAs were used to construct a ceRNA network that contained 96 nodes and 119 edges **(Figure 10)**.

### 3.11 Consistency of *HES4* expression trend in TCGA-BLCA and GSE121711

**Figure 11:**
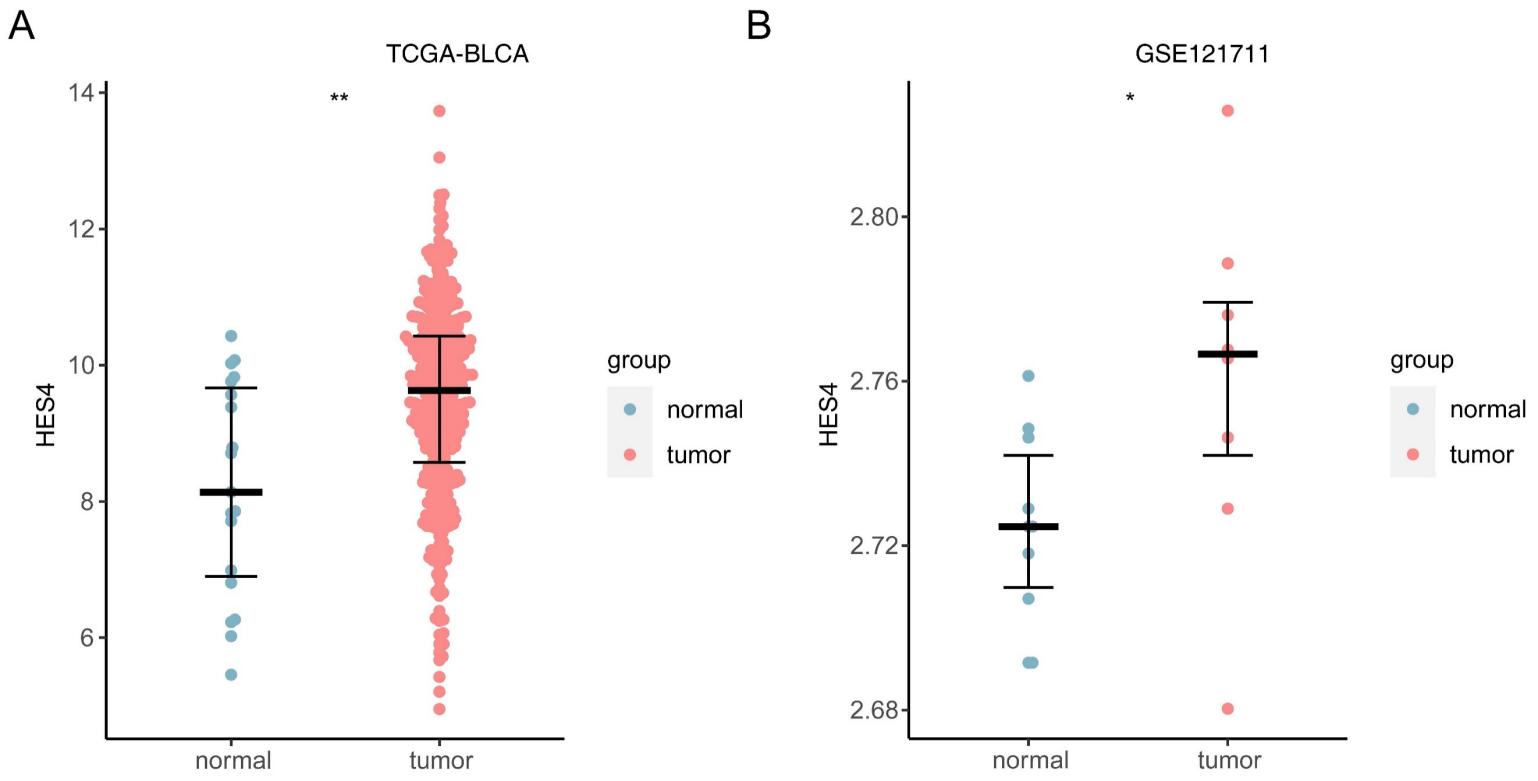
Expression of *HES4* in public datasets. **A** TCGA-BLCA. **B** GSE121711. *p< 0.05, **p< 0.01.

In both TCGA and GSE121711 datasets, *HES4* was up-regulated in cancer samples (TCGA: *P* < 0.01; GSE121711: *P* < 0.05; **Figure 11A-B**).

## 4. Discussion

Although though the majority of BLCA patients present with NMIBC at the first diagnosis, the prognosis progressively deteriorates on account of the highly recurrent and invasive nature of BLCA. Many genes associated with BLCA prognosis have been reported, along with genes whose expression and/or mutation can be used for diagnosis 30-32, but to date, there is still a lack of sufficiently sensitive and accurate molecular markers of BLCA to expedite diagnosis in order to both reduce the requirement for invasive investigations and to ensure timely provision of treatment. In this study, we identified *HES4* as being causally associated with BLCA using transcriptomics and MR. *HES4* could both serve as an independent prognostic factor and play a crucial role in tumor progression, molecular interactions, and immune response processes.

Following a similar approach, Li et al. 33 obtained a systemic lupus erythematosus (SLE)-related gene signature (SLEscore) for predicting the prognosis of patients with coexistent SLE and breast cancer, with 3- and 5-year AUCs of 0.81 and 0.91, respectively 34,35, indicating that a combined transcriptome/MR analysis can lead to more robust outcomes than transcriptome analysis alone. In the current study, we established a nomogram to predict the 1 -, 3 -, and 5-year survival rates of BLCA patients by combining age, stage, and *HES4* expression. Our results indicated this nomogram was most effective at predicting the 1-year survival rate of BLCA patients. The failure of the model to predict 3- and 5-year survival rates may imply that the development of BLCA is multifactorial.

*HES4*, a Hes family BHLH transcription factor, can repress the expression of target genes by recruiting co-repressors 36. In the hematopoietic system, as a downstream gene of the Notch pathway, *HES4* promoted early T-lineage differentiation and induced T-cell development, while repressing the development of NK and myeloid cells 36. Our immune infiltration analysis was consistent with this, with the expression level of *HES4* negatively correlated with macrophages/monocytes and NK cells, and positively correlated with some CD4^+^ T cells. During tumorigenesis, high expression of *HES4* can increase the invasiveness of osteosarcoma cells and inhibit calcium deposition, and it is negatively correlated with OS 37. High expression of the *HES4* gene was also reported to be significantly correlated with activating mutations to Notch genes in breast cancers, which resulted in poorer prognosis 38. In our study, the expression of *HES4* was significantly increased in BLCA patients, and the TMB level was higher in the group with high expression of *HES4*, yet the results of the multivariable cox regression analysis demonstrated that *HES4* may exert quite distinctive effects in different developmental stages of BLCA. GSEA did not identify enrichment of the Notch pathway. However, several other pathways were enriched, including pathways related to cellular senescence, energy metabolism, and neuronal diseases, suggesting that there may be additional important regulatory mechanisms beyond the Notch pathway in BLCA, which should be investigated in future experimental studies.

AZD6482 is a phosphoinositide 3-kinase β (PI3Kβ) inhibitor 39. The PI3K/Akt pathway and the MAPK pathway extensively interact in BLCA 40, low expression of *HES4* was associated with the MAPK pathway in GSEA analysis, and there was a positive correlation between the expression of *HES4* and the IC_50_ value of AZD6482 in the GDSC database. Overall, these data suggest that the BLCA patients with low *HES4* expression may be more sensitive to AZD6482 treatment.

Numerous studies have reported that the tumor microenvironment plays an integral role in tumor progression, including in BLCA 41-43. Parizi et al. demonstrated that tumor-infiltrating immune cells were strongly associated with the survival and biological behavior of BLCA patients 44. To explore the immune microenvironment in BLCA and its relationship with *HES4*, we performed an immune infiltration analysis. The proportions of T-cells, monocytes, macrophages/monocytes, and neutrophils not only differed significantly between BLCA and normal samples, but were also strongly correlated with* HES4*. It has been reported that *ETV4* promotes lymphangiogenesis and lymph node metastasis in BLCA by regulating tumor-associated neutrophil infiltration; lymph node metastasis is the main cause of death in BLCA 45. Huang et al. demonstrated that* HSF1* was positively correlated with BLCA lymphatic metastasis and poor prognosis, and increased the infiltration of tumor-associated macrophages, especially M2 macrophages. We believe that the differences in immune cell infiltration in BLCA may be related to* HES4*, but the molecular mechanisms involved need to be explored in further experiments.

To our knowledge, this is the first study to use transcriptomics and MR analysis to identify a possible causal relationship between *HES4* expression and the occurrence and progression of BLCA. We constructed a nomogram to predict the 1-year survival of patients combining age, stage, and *HES4* expression. We preliminarily unveiled the potential complex mechanism of *HES4* in BLCA using enrichment analysis, immune infiltration analysis, and construction of a ceRNA network. We used the GDSC database and molecular docking to identify AZD6482 as a drug potentially targeting *HES4*, providing a novel candidate drug for the treatment of BLCA. This has important clinical implications for the early diagnosis, treatment, and prognosis of BLCA.

There are some limitations to our study. Firstly, this is an observational study and causality cannot be confirmed. Although MR analysis can provide some evidence of causality, experimental validation is still required. Secondly, although we used transcriptome analysis to investigate the mechanisms by which *HES4* might promote BLCA, further experimental studies are needed to verify these mechanisms. Finally, although a potential target drug was identified, its efficacy and safety still need to be validated in animal models and clinical trials.

Overall, this study provides new clues to our understanding of the relationship between *HES4* and BLCA and offers new directions for the treatment of BLCA.

## Conclusions

*HES4* was identified as a diagnostic and prognostic biomarker for BLCA, and a nomogram combining age, stage, and *HES4* expression predicts the 1-year survival of patients. We also identified AZD6482 as a potential drug for targeted treatment of BLCA, which may be significant for clinical decision-making in BLCA patients.

## Supplementary Material

Supplementary figures and tables.

## Figures and Tables

**Table 1:** Sensitivity analysis based on the heterogeneity test.

id.exposure	id.outcome	outcome	exposure	method	Q	Q_df	Q_pval
eqtl-a-ENSG00000106133	ieu-b-4874	Bladder cancer || id:ieu-b-4874	ENSG00000106133 || id:eqtl-a-ENSG00000106133	MR Egger	8.78E-01	1	0.348787793
eqtl-a-ENSG00000106133	ieu-b-4874	Bladder cancer || id:ieu-b-4874	ENSG00000106133 || id:eqtl-a-ENSG00000106133	Inverse variance weighted	9.59E-01	2	0.619175929
eqtl-a-ENSG00000120708	ieu-b-4874	Bladder cancer || id:ieu-b-4874	ENSG00000120708 || id:eqtl-a-ENSG00000120708	MR Egger	10.0967612	15	0.813609607
eqtl-a-ENSG00000120708	ieu-b-4874	Bladder cancer || id:ieu-b-4874	ENSG00000120708 || id:eqtl-a-ENSG00000120708	Inverse variance weighted	1.09E+01	16	0.815708412
eqtl-a-ENSG00000154928	ieu-b-4874	Bladder cancer || id:ieu-b-4874	ENSG00000154928 || id:eqtl-a-ENSG00000154928	MR Egger	5.55E+00	8	0.697669938
eqtl-a-ENSG00000154928	ieu-b-4874	Bladder cancer || id:ieu-b-4874	ENSG00000154928 || id:eqtl-a-ENSG00000154928	Inverse variance weighted	5.61E+00	9	0.778223064
eqtl-a-ENSG00000168062	ieu-b-4874	Bladder cancer || id:ieu-b-4874	ENSG00000168062 || id:eqtl-a-ENSG00000168062	MR Egger	1.147019255	1	0.284173994
eqtl-a-ENSG00000168062	ieu-b-4874	Bladder cancer || id:ieu-b-4874	ENSG00000168062 || id:eqtl-a-ENSG00000168062	Inverse variance weighted	1.46E+00	2	0.482399521
eqtl-a-ENSG00000188290	ieu-b-4874	Bladder cancer || id:ieu-b-4874	ENSG00000188290 || id:eqtl-a-ENSG00000188290	MR Egger	2.96E-03	1	0.956599707
eqtl-a-ENSG00000188290	ieu-b-4874	Bladder cancer || id:ieu-b-4874	ENSG00000188290 || id:eqtl-a-ENSG00000188290	Inverse variance weighted	1.54E+00	2	0.463305485
eqtl-a-ENSG00000221866	ieu-b-4874	Bladder cancer || id:ieu-b-4874	ENSG00000221866 || id:eqtl-a-ENSG00000221866	MR Egger	1.868367911	1	0.171662122
eqtl-a-ENSG00000221866	ieu-b-4874	Bladder cancer || id:ieu-b-4874	ENSG00000221866 || id:eqtl-a-ENSG00000221866	Inverse variance weighted	1.92E+00	2	0.383487672
eqtl-a-ENSG00000240288	ieu-b-4874	Bladder cancer || id:ieu-b-4874	ENSG00000240288 || id:eqtl-a-ENSG00000240288	MR Egger	1.18E+00	2	0.554819131
eqtl-a-ENSG00000240288	ieu-b-4874	Bladder cancer || id:ieu-b-4874	ENSG00000240288 || id:eqtl-a-ENSG00000240288	Inverse variance weighted	2.45E+00	3	0.484700371
eqtl-a-ENSG00000241015	ieu-b-4874	Bladder cancer || id:ieu-b-4874	ENSG00000241015 || id:eqtl-a-ENSG00000241015	MR Egger	0.140335832	2	0.932237269
eqtl-a-ENSG00000241015	ieu-b-4874	Bladder cancer || id:ieu-b-4874	ENSG00000241015 || id:eqtl-a-ENSG00000241015	Inverse variance weighted	1.58E+00	3	0.664332184

**Table 2:** Sensitivity analysis based on the horizontal pleiotropy test.

id.exposure	id.outcome	outcome	exposure	egger_intercept	se	pval
eqtl-a-ENSG00000106133	ieu-b-4874	Bladder cancer || id:ieu-b-4874	ENSG00000106133 || id:eqtl-a-ENSG00000106133	-7.10E-05	0.000249604	0.82361746
eqtl-a-ENSG00000120708	ieu-b-4874	Bladder cancer || id:ieu-b-4874	ENSG00000120708 || id:eqtl-a-ENSG00000120708	5.08E-05	5.67E-05	0.38472654
eqtl-a-ENSG00000154928	ieu-b-4874	Bladder cancer || id:ieu-b-4874	ENSG00000154928 || id:eqtl-a-ENSG00000154928	-1.93E-05	7.79E-05	0.81020401
eqtl-a-ENSG00000168062	ieu-b-4874	Bladder cancer || id:ieu-b-4874	ENSG00000168062 || id:eqtl-a-ENSG00000168062	-0.00061497	0.001181128	0.69439614
eqtl-a-ENSG00000188290	ieu-b-4874	Bladder cancer || id:ieu-b-4874	ENSG00000188290 || id:eqtl-a-ENSG00000188290	-0.000222855	0.000179828	0.43223463
eqtl-a-ENSG00000221866	ieu-b-4874	Bladder cancer || id:ieu-b-4874	ENSG00000221866 || id:eqtl-a-ENSG00000221866	-0.000167165	0.001037243	0.89827553
eqtl-a-ENSG00000240288	ieu-b-4874	Bladder cancer || id:ieu-b-4874	ENSG00000240288 || id:eqtl-a-ENSG00000240288	-0.00021807	0.000193497	0.3767828
eqtl-a-ENSG00000241015	ieu-b-4874	Bladder cancer || id:ieu-b-4874	ENSG00000241015 || id:eqtl-a-ENSG00000241015	-0.000197497	0.0001647	0.35327642

**Table 3:** Results for independent prognostic univariate COX analysis.

variable	coef	HR	HR.95L	HR.95H	pvalue
N	0.462116395	1.587430062	1.345801715	1.872440919	4.13E-08
STAGE	0.610093568	1.840603612	1.477634912	2.292732548	5.21E-08
age	0.035464194	1.036100549	1.018794898	1.05370016	3.68E-05
HES4	-0.187302277	0.829193053	0.745891558	0.921797695	0.000525474
gender	-0.122877782	0.884371742	0.622876769	1.25564705	0.492041845

**Table 4:** Results for independent prognostic multivariate COX analysis.

id	coef	HR	HR.95L	HR.95H	pvalue
age	0.033528694	1.034097116	1.016531265	1.051966507	1.25E-04
STAGE	0.579303126	1.78479422	1.430066689	2.227511789	2.99E-07
HES4	-0.18997349	0.826981057	0.74121574	0.922670191	6.72E-04
