# Supplementary figures and images for: HES4 is a potential biomarker for bladder cancer: a Mendelian randomization study
Source: J Cancer. 2024 Jan 21;15(6):1624–41. doi: 10.7150/jca.92657 (PMC10869984; doi:10.7150/jca.92657)

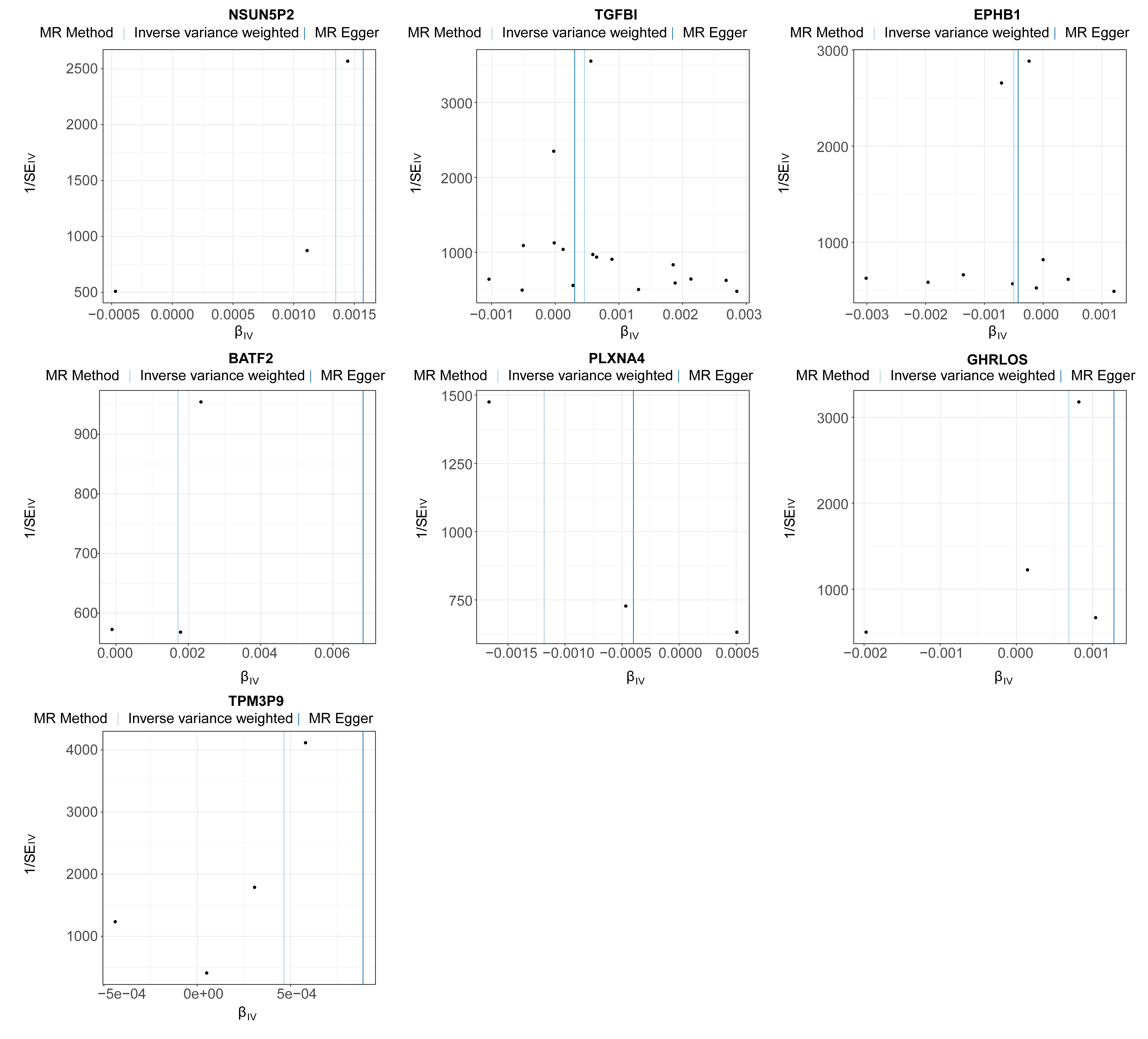

Supplement: Supplementary file 1 — Supplementary figures and tables. [file jcav15p1624s1.zip › Figure S3.tif]

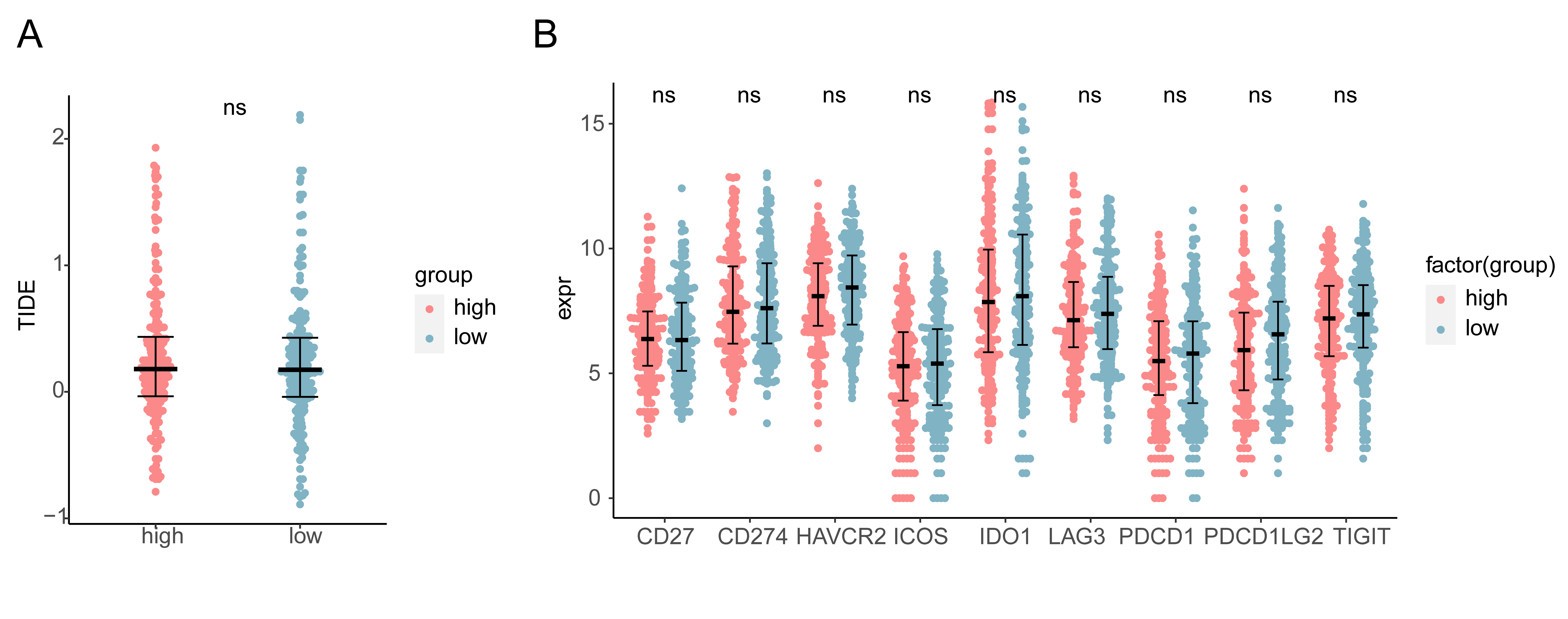

Supplement: Supplementary file 1 — Supplementary figures and tables. [file jcav15p1624s1.zip › Figure S5.tif]
